# Supplementary material for: Drug Repurposing for Influenza Virus Polymerase Acidic (PA) Endonuclease Inhibitor
Source: Molecules. 2021 Dec 2;26(23):7326. doi: 10.3390/molecules26237326 (PMC8659148; doi:10.3390/molecules26237326)
Supplement: Supplementary file 1 [file molecules-26-07326-s001.zip › molecules-1468825-supplementary.pdf]

molecules

MDPI

Supporting information

Drug Repurposing for Influenza Virus Polymerase Acidic (PA) Endonuclease Inhibitor

Xin Meng [1,2] and Ye Wang [1,*]

[1] School of Life Science, Jilin University, No. 2699 Qianjin Street, Changchun 130012, China
[2] School of Life Science, Jilin Normal University, Siping 136000, China; 18624395651@163.com
* Correspondence: wangye0106@jlu.edu.cn; Tel.: +86-431-8515-5249

Table S1. The properties and energy score of the potential PA_N inhibitors.

Chemical name	Structure	Energy* (kcal/mol)	M_{wt}	Chemical Formula	Summary
Saquinavir	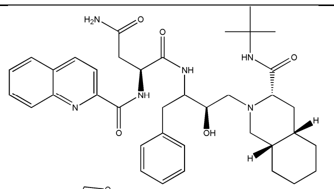	−10.8	670.8	C38H50N6O5	HIV protease inhibitor used in combination with other antiretroviral agents for the treatment of HIV-1 with advanced immunodeficiency.
Teniposide	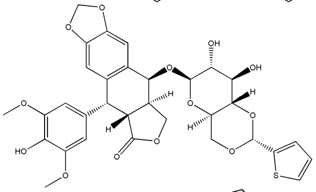	−10.1	656.7	C32H32O13S	Cytotoxic drug used as an adjunct for chemotherapy induction in the treatment of refractory childhood acute lymphoblastic leukemia.
Conivaptan	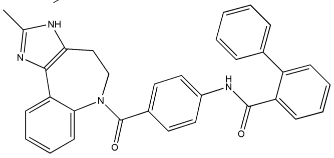	−10	498.6	C32H26N4O2	Antidiuretic hormone inhibitor used to raise serum sodium levels.
Lifitegrast	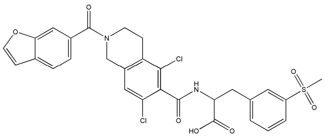	−10	615.5	C29H24Cl2N2O7S	Medication used to treat dry eye disease.
Simeprevir	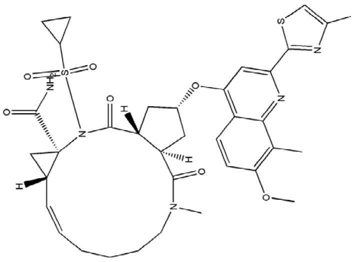	−9.9	749.9	C38H47N5O7S2	Direct-acting antiviral agent that inhibits HCV NS3/4A protease to treat chronic hepatitis C virus (HCV) infection in adults with HCV genotype 1 or 4.
Nilotinib	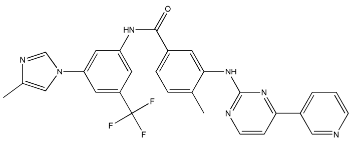	−9.8	529.5	C28H22F3N7O	Kinase inhibitor used for the chronic phase treatment of Chronic Myeloid Leukemia (CML) that is Philadelphia chromosome-positive and for the treatment of CML that is resistant to therapy containing imatinib.

Rifaximin	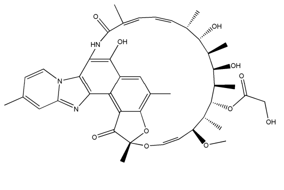	−9.8	785.9	C43H51N3O11	Rifamycin-based non-systemic antibiotic used for the treatment of gastrointestinal bacterial infections, such as traveler's diarrhea and irritable bowel syndrome, and reduction in overt hepatic encephalopathy recurrence in adults.
Dutasteride	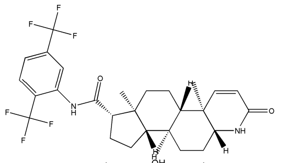	−9.7	528.5	C27H30F6N2O2	Antiandrogenic compound that is used for the treatment of symptomatic benign prostatic hyperplasia (BPH) in adult males by inhibiting 5-alpha reductase.
Etoposide	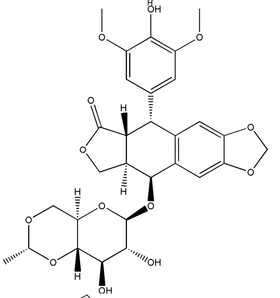	−9.7	588.6	C29H32O13	Podophyllotoxin derivative used to treat testicular and small cell lung tumors.
Ergotamine	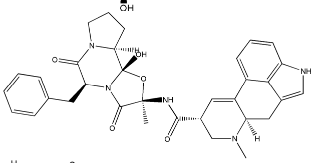	−9.6	581.7	C33H35N5O5	Alpha-1 selective adrenergic agonist vasoconstrictor used to treat migraines with or without aura and cluster headaches.
Lurasidone	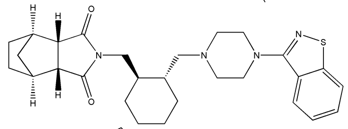	−9.5	492.7	C28H36N4O2S	Atypical antipsychotic used to treat schizophrenia and depressive episodes associated with bipolar I disorder.
Dihydroergotamine	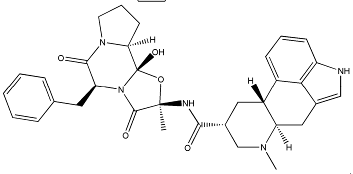	−9.5	583.7	C33H37N5O5	Ergot alkaloid used in the acute treatment of migraine headaches and cluster headaches.
Naldemedine	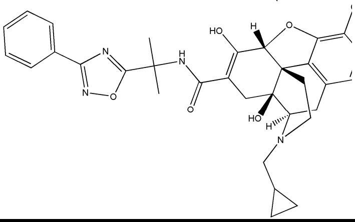	−9.5	570.6	C32H34N4O6	Opioid antagonist used to treat opioid-induced constipation.

* Affinity binding energy of virtual screening results by AutoDock vina program.

Table S2. The properties and energy score of the potential PAN-I38T endonuclease inhibitors.

Chemical name	Structure	Energy* (kcal/mol)	M_{wt}	Chemical Formula	Summary
Saquinavir	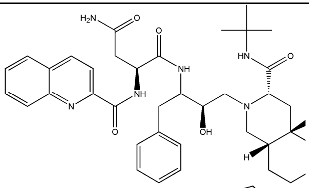	−10.3	670.8	C38H50N6O5	HIV protease inhibitor used in combination with other antiretroviral agents for the treatment of HIV-1 with advanced immunodeficiency.
Conivaptan	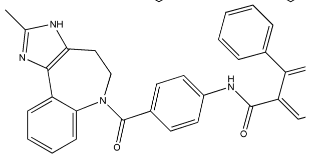	−10.1	498.6	C32H26N4O2	Antidiuretic hormone inhibitor used to raise serum sodium levels.
Nilotinib	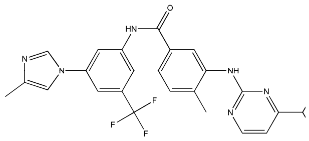	−10	529.5	C28H22F3N7O	Kinase inhibitor used for the chronic phase treatment of Chronic Myeloid Leukemia (CML) that is Philadelphia chromosome-positive and for the treatment of CML that is resistant to therapy containing imatinib.
Teniposide	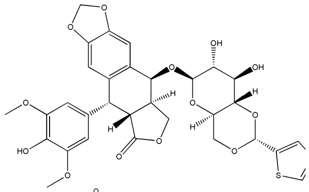	−9.9	656.7	C32H32O13S	Cytotoxic drug used as an adjunct for chemotherapy induction in the treatment of refractory childhood acute lymphoblastic leukemia.
Lifitegrast	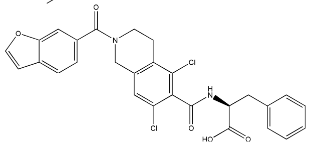	−9.9	615.5	C29H24Cl2N2O7S	Medication used to treat dry eye disease.
Naldemedine	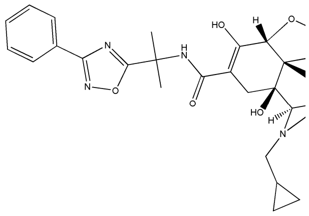	−9.9	570.6	C32H34N4O6	Opioid antagonist used to treat opioid-induced constipation.
Simeprevir	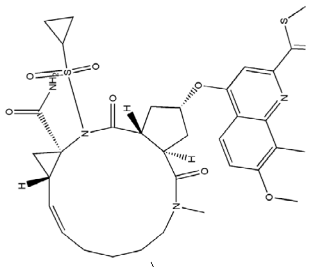	−9.9	749.9	C38H47N5O7S2	Direct-acting antiviral agent that inhibits HCV NS3/4A protease to treat chronic hepatitis C virus (HCV) infection in adults with HCV genotype 1 or 4.
Rifaximin	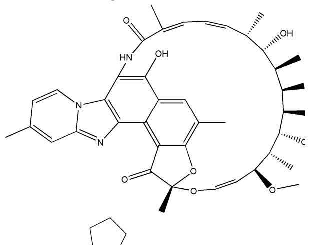	−9.9	785.9	C43H51N3O11	Rifamycin-based non-systemic antibiotic used for the treatment of gastrointestinal bacterial infections, such as traveler's diarrhea and irritable bowel syndrome, and reduction of overt hepatic encephalopathy recurrence in adults.
Ergotamine	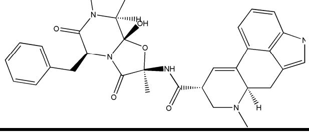	−9.7	581.7	C33H35N5O5	Alpha-1 selective adrenergic agonist vasoconstrictor used to treat migraines with or without aura and cluster headaches.

* Affinity binding energy of virtual screening results by AutoDock vina program.

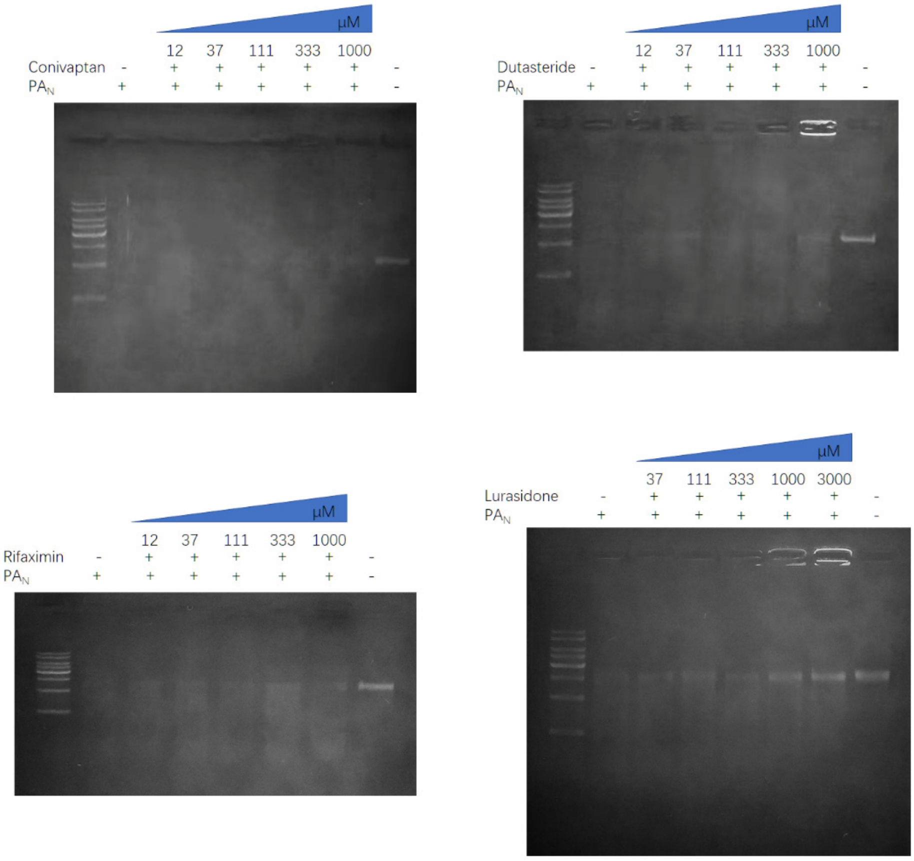

Figure S1. Compounds inhibition for the endonuclease activity of PAN. Different concentrations of conivaptan (0–333μM), dutasteride (0–1000μM), rifaximin (0–1000μM), and lurasidone (0–3000μM) incubated with the PA and substrate ssDNA for 1 h at 37 °C.

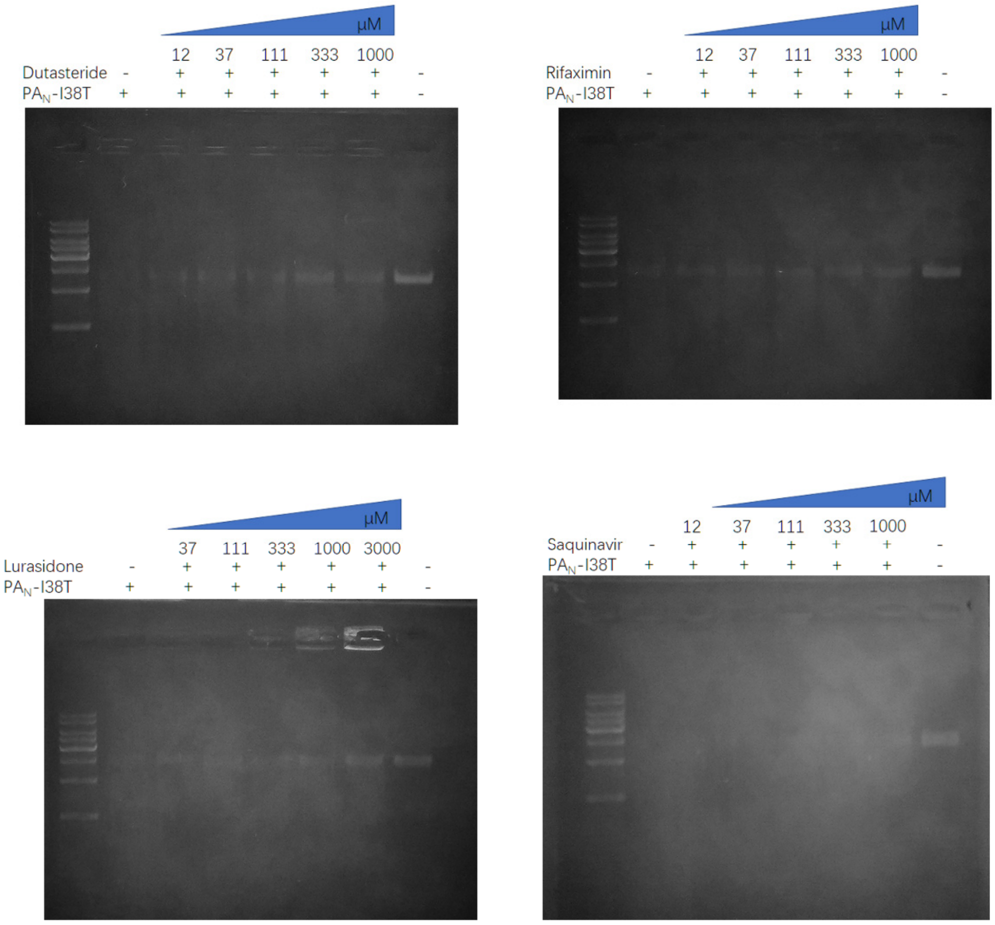

Figure S2. Compounds inhibition for the endonuclease activity of PAN-I38T. Different concentrations of dutasteride (0–1000μM), rifaximin (0–1000μM), saquinavir (0–1000μM), and lurasidone (0–3000μM) incubated with the PAN-I38T and substrate ssDNA for 1h at 37 °C.
